# Hearing Health Monitoring in Adults Living with Diabetes: A Scoping Review to Inform Integrated Care Pathways

**DOI:** 10.3390/audiolres16050134

**Published:** 2026-09-10

**Authors:** Grace Malheiro, Christine Meston, Chris Allan, Jana N. Bataineh, Paleesa Kapoor, Robin O’Hagan, Danielle Glista

**Affiliations:** 1 Health and Rehabilitation Sciences, Western University, London, ON N6G 1H1, Canada; gmalheir@uwo.ca (G.M.); jbataine@uwo.ca (J.N.B.); 2 The National Centre for Audiology, Western University, London, ON N6G 1H1, Canada; cmeston@uwo.ca (C.M.); callan3@uwo.ca (C.A.);; 3 School of Communication Sciences and Disorders, Western University, London, ON N6G 1H1, Canada; 4 H.A. Leeper Speech and Hearing Clinic, Western University, London, ON N6G 1H1, Canada; 5 Faculty of Science, Western University, London, ON N6A 5B7, Canada; pkapoo22@uwo.ca

**Keywords:** hearing loss, diabetes, audiology, interdisciplinary care, adults

## Abstract

**Objectives**: Diabetes is a significant risk factor for hearing loss, highlighting the need for evidence-informed hearing health monitoring. Undiagnosed and untreated hearing loss can impair communication, reduce participation in daily activities, and diminish quality of life. This scoping review synthesized the current evidence on hearing loss screening and assessment in adults living with diabetes and identified knowledge gaps to inform the development of an evidence-informed hearing health monitoring framework. **Design**: A scoping review was conducted in accordance with the Joanna Briggs Institute methodology. Seven electronic databases were searched for peer-reviewed studies reporting primary data on the relationship between diabetes and hearing loss. **Results**: A total of 201 studies met the inclusion criteria. The literature represented a global evidence base, with studies conducted across diverse settings, including hospitals, community and diabetes clinics, schools, audiology clinics, outpatient clinics, and research centres. Pure-tone audiometry was the most frequently used assessment, followed by electrophysiological measures and otoacoustic emissions. Fewer than half of the studies provided recommendations for hearing screening, assessment, or monitoring, and only six advocated for early referral to hearing healthcare services. **Conclusions**: Despite growing evidence linking diabetes and hearing loss, important gaps remain in recommendations for diagnostic assessment, hearing health monitoring, referral pathways, and interdisciplinary integration within diabetes care. The findings of this review provide a foundation for developing an evidence-informed hearing health monitoring framework and support the integration of hearing health into coordinated person-centred diabetes care pathways.

## 1. Introduction

Diabetes mellitus (DM), affecting approximately 11% of Canadian adults, is a significant risk factor for the development of retinopathy, neuropathy, nephropathy, and cardiovascular diseases [1,2]. More recently, hearing loss (HL) has been recognized as a complication of DM, with established evidence demonstrating an association between DM and auditory dysfunction. While the more established complications of DM are routinely monitored as part of diabetes management, hearing loss remains underrecognized and has yet to be incorporated into standard clinical care pathways [3,4]. Evidence suggests that HL may develop at a younger age in individuals with DM and is about twice as prevalent among people living with diabetes compared to those without the condition, even after adjusting for age and other risk factors for HL [5,6,7]. A recent systematic review and meta-analysis estimated that nearly one in four adults living with DM has a moderate-to-severe hearing loss (≥40 dB HL) [8]. Despite this substantial burden, hearing health has yet to be routinely incorporated into diabetes care, highlighting an important gap between the available evidence and current clinical practice [8].

The need to monitor and manage hearing health in individuals living with DM is increasingly recognized by national and international health organizations. Diabetes Canada [9] acknowledges that individuals living with DM are at an increased risk of HL while the World Health Organization [10] continues to consistently advocate for hearing screening for this population. Recommendations have also been proposed to inform and support early detection and management of HL in individuals living with DM, using current evidence-based audiological measurement tools [7]. Comprehensive audiological awareness, monitoring protocols, and guidance for integrating hearing care into routine diabetes management remain lacking.

Undiagnosed and untreated HL in adulthood extends beyond a sensory impairment. It adversely affects communication, limits participation in daily activities, and contributes to social isolation, loneliness, and reduced quality of life [11,12]. Reduced social engagement, poorer mental well-being, and decreased physical activity can also increase the risk of cognitive decline and dementia [13]. As the Canadian population continues to increase, the prevalence of DM and its associated personal, societal, and healthcare costs are expected to increase as well [14]. These challenges underscore the need to identify effective resources, strategies, and models of care to support early identification, monitoring, and management of HL among adults with DM. The purpose of this scoping review is to gather, summarize, and synthesize the peer-reviewed literature on early detection, assessment, and monitoring of hearing loss in adults living with diabetes, as well as on primary care integration pathways to support their care. A secondary aim was to use the synthesized evidence to propose a practical framework for integrating hearing health into routine diabetes management.

## 2. Methods

This review was undertaken following the Joanna Briggs Institute (JBI) methodology for scoping reviews and is presented in accordance with the Preferred Reporting Items for Systematic reviews and Meta-Analyses extension for Scoping Reviews (PRISMA-ScR) guidance and checklist [15].

### 2.1. Protocol

The PRISMA-ScR checklist is provided in the Supplementary Material (File S1). Ethics approval was not required for this study. Scoping reviews are exempt from research ethics review when they rely on secondary, anonymized data.

### 2.2. Eligibility Criteria

Studies were included using the following criteria: (a) peer reviewed sources; (b) published or accessible in English; (c) electronic full-text available; (d) primary data; (e) including adult participants aged 18 years or older; (f) DM as the primary disease of interest, not only a risk factor for another disease; (g) hearing status identified through an auditory system assessment; and (h) relationship between DM and HL. Studies published in languages other than English were excluded due to the research team’s language limitations. Grey literature was excluded based on a preliminary search indicating limited relevant guidance. The review therefore focused on primary research evidence to inform the development of future guidance.

### 2.3. Information Sources

Seven databases were searched: MEDLINE(R) ALL (Ovid), Cochrane Central Register of Controlled Trials, Embase and Embase Classic (Ovid), APA PsycInfo (Ovid), CINAHL (EBSCO), Scopus, and Web of Science. Databases were selected in consultation with a research librarian at Western University to maximize the comprehensiveness of the search. No limits were placed on the study context or the publication date of included studies. The search was completed in April 2026 across all seven databases.

### 2.4. Search Terms

A list of preliminary search terms was created by five members of the research team (CA, CM, DG, JB, PK). The search terms were refined with input from a research and scholarly communications librarian to incorporate subject headings, keywords (e.g., diabetes, hearing loss), and indexing terms such as Medical Subject Headings. Appendix A details a full set of search terms used in the Ovid MEDLINE database, which were replicated and modified for the remaining databases.

### 2.5. Selection of Sources of Evidence

A three-step search strategy was employed in the scoping review protocol: an initial search, modification of the search strategy, and screening processes. Step one involved an initial search limited to two online databases, conducted by one member of the research team (JB), to determine the suitability of the search terms and the availability of existing evidence on the topic. Search terms were modified to ensure that the findings were comprehensive and aligned with the objectives of the scoping review following this step. Step two identified articles through analysis of text words in the titles and abstracts, and index terms from the initial search, followed by a comprehensive search across all included databases using the refined keywords and indexed terms. All records were imported into Covidence, an online systematic review management platform, where duplicate articles were automatically removed [16]. A pilot screening was conducted following the JBI protocol (Section 10.2.6, Source of Evidence Selection), in which five team members (CA, CM, DG, JB, PK) reviewed 25 randomly selected articles to ensure consistent application of the eligibility criteria. Any disagreements over the eligibility of evidence sources for inclusion in the review were discussed and resolved in team meetings during the screening phases. All research team members independently conducted title and abstract screening using Covidence. Team meetings (CA, CM, DG, GM, JB, PK, RO) were held to achieve consensus when disagreements arose around the inclusion of specific studies. Evidence that passed the title/abstract screening phase was then moved to the full-text stage, where two members independently screened against the inclusion criteria. Evidence from the full-text stage that met the inclusion criteria was included and extracted.

### 2.6. Data Charting Process and Data Items

A draft data charting table to guide data extraction (Supplement File S2) was developed by the research team using Microsoft Excel (Version 16.64). All members of the research team were involved in the data extraction phase. To ensure consistency in data extraction and agreement on extraction components, four members (DG, CA, CM, JB) piloted the extraction form with two randomly selected studies. The final data charting table included components such as study design, participant details, type of audiological testing tools, healthcare professional roles, and study findings. Data extraction for each article was completed by at least two research team members. Data extraction was independently conducted within Covidence, and a third team member completed the consensus step to ensure alignment of data extracted from each article. If disagreements arose, they were discussed during regularly scheduled team meetings. Data extraction was conducted iteratively, with categories added or modified in consultation with the full research team to align with the review objectives.

## 3. Results

**Figure 1:**
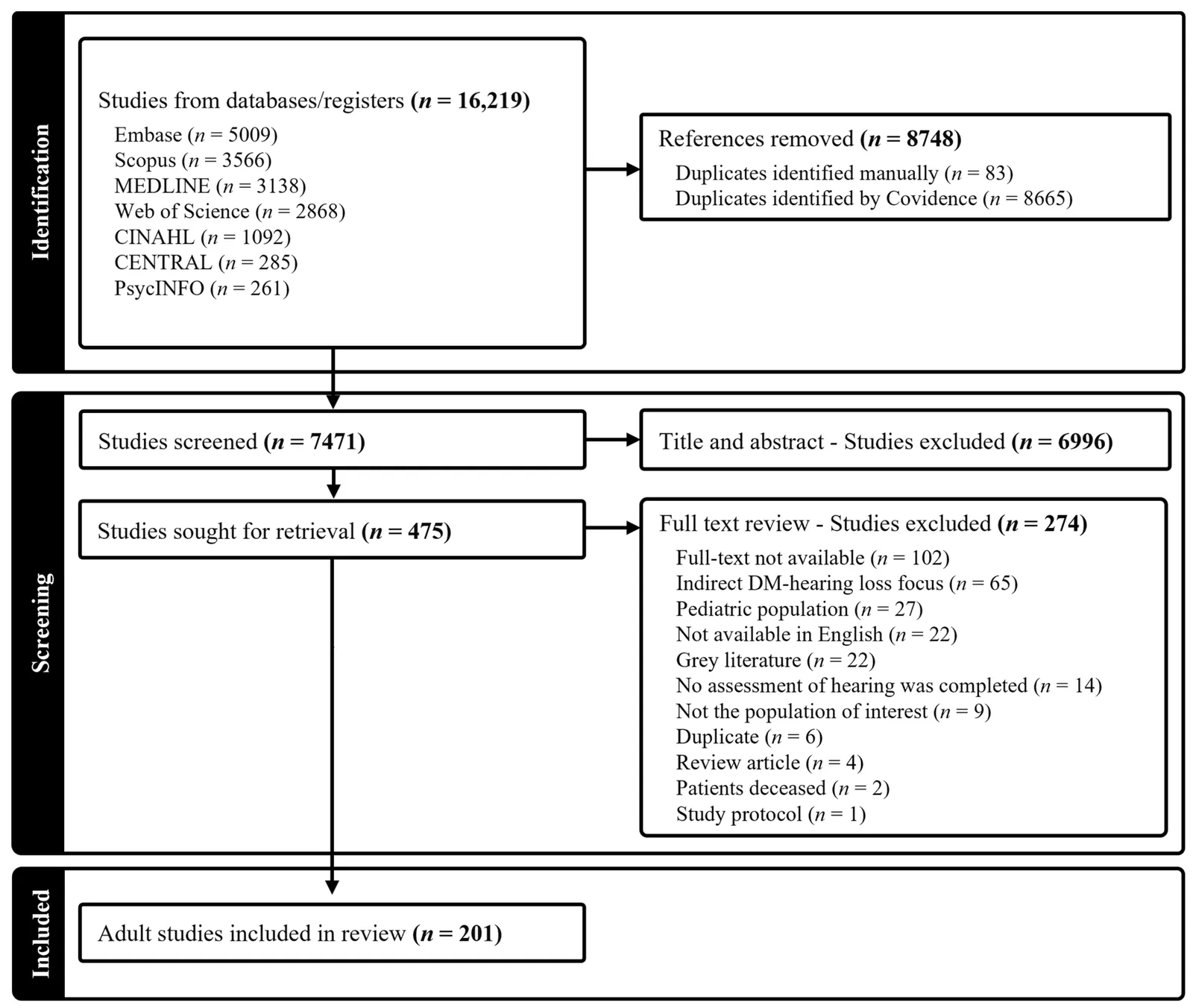
PRISMA-ScR Flow Diagram.

A total of 16,219 studies were identified, of which 8748 were duplicates. The title and abstract screening resulted in the removal of 6996 studies that did not meet the eligibility criteria, followed by the removal of 274 studies during full-text review. During the data extraction phase, 201 adult-focused studies were identified. The PRISMA-ScR flow diagram (Figure 1) summarizes the process for evidence inclusion as a result of this review. The final set of included articles is summarized in Appendix B according to study design, aims, participant age, healthcare providers involved in audiological care, assessment tools, and recommendations. Articles were assessed using the Oxford Centre for Evidence-Based Medicine (CEBM) for levels of evidence [17].

### 3.1. Global Representation and Timeline

The literature in this scoping review spanned more than six decades, from 1974 to 2026. The included studies represented a diverse international body of literature, with India contributing the largest number of publications (*n* = 76). By continent, most studies originated from Asia (*n* = 128), followed by Europe (*n* = 28), North America (*n* = 24), Africa (*n* = 11), South America (*n* = 7), and Australia (*n* = 3).

### 3.2. Diabetes Classification, Age Classification, and Duration of Diabetes

Most studies exclusively investigated T2DM (*n* = 103), with a small number studying only T1DM (*n* = 21); some studies assessed both T1DM and T2DM (*n* = 21). Approximately one-quarter of the studies did not specify the type of DM (*n* = 56). Reporting of participant age was incomplete across studies, with many failing to report either the age range or the mean age. Among studies that provided age-related information, participants were at least 18 years of age, with reported upper age limits extending to ≥70 years; however, the true upper age range across all included studies could not be determined. Reporting of participant characteristics was incomplete across the included studies. Among studies that reported these data, 131 reported the mean age of participants living with DM, 88 reported the mean or range of diabetes duration, and 20 reported the time of diabetes onset.

### 3.3. Healthcare Professionals and Professional Settings

Reporting of the healthcare professionals involved in the identification of HL was limited across the included studies. Of the 201 studies included in this review, only 11 specified which professional was involved in the care of the patient. Professionals included one audiologist, four physicians, one research physician, one general practitioner, one primary care practitioner, one internist, one otologist, two ophthalmologists, and one unspecified healthcare provider.

The professional settings involved in HL identification were often reported at a broad departmental level, lacking contextual detail. Hospitals were the most frequently reported location (*n* = 106). Additional settings included: within the research study (*n* = 32), school (*n* = 12), DM-specific setting (*n* = 11), community-based setting (*n* = 6), general healthcare centre (*n* = 5), audiology clinic (*n* = 4), and outpatient clinic (*n* = 2). In several studies, the setting was not reported (*n* = 23).

### 3.4. Case History and Patient-Reported Outcome Measures

Some studies collected case history information focused on demographics and medical information (*n* = 132); however, fewer studies employed patient-reported outcome measures or questionnaires to assess the impact of potential auditory system damage on daily functioning (*n* = 23). Of the questionnaires mentioned, the Michigan Neuropathy Screening Instrument was most frequently used to screen for diabetic peripheral neuropathy (*n* = 5) [18]. Other questionnaires focused on cognitive impairment, noise exposure, dizziness, and psychosocial impact.

### 3.5. Comorbidities and Risk Factors

Information on the presence of comorbidities alongside diabetes was mentioned in approximately half of the studies (*n* = 121). The most common comorbidities included retinopathy (*n* = 61), hypertension (HTN; *n* = 58), neuropathy (*n* = 50), and nephropathy (*n* = 26). Braffett and colleagues [19] reported that risk factors such as retinopathy, kidney disease, neuropathy, and a history of HTN, among others, were significantly associated with a higher likelihood of speech-frequency hearing loss. Studies examining comorbidities yielded heterogeneous findings: some reported significant associations with hearing outcomes, while others found none.

Some studies examined poor glycemic and metabolic control as a risk factor for HL. Esubalew and colleagues [20] reported that glycemic control was significantly associated with SNHL, defined as an inability to perceive sounds at 25 dB or lower in one or both ears. HbA1c levels, an indicator of long-term glycemic control, were often reported to be higher among individuals living with DM. Krishnappa and Naseeruddin [21], showed a positive relation between HbA1c and the severity of HL. As glycemic control deteriorated, the severity of HL increased. When glycemic control was poor, most cases presented with either moderate or moderately severe HL [21]. Additionally, hearing loss was observed across a range of frequencies, with a greater degree of loss noted among individuals living with DM compared to non-diabetic individuals, particularly at higher frequencies [21].

The duration of DM was inconsistently reported across studies, with some providing wide-ranging values such as 0.1 to 30.0 years [22], while others did not report duration. Among studies that included duration as a variable, results were mixed as to whether it was a risk factor for HL. Dosemane and colleagues [23] grouped duration of DM into <5 and >5 years (no mean was provided). They noted that the duration of DM did not statistically impact the severity of HL. J. Li and colleagues [24] indicated a possible correlation between the duration of DM, glycemic control and the incidence of high frequency HL. Specifically, when comparing groups with T2DM for <10 or ≥10 years, no significant difference in HL was observed between the two groups, but at higher frequencies, significant differences in HL were observed among patients according to both disease duration and HbA1c levels.

### 3.6. Hearing Assessment Tools

**Figure 2:**
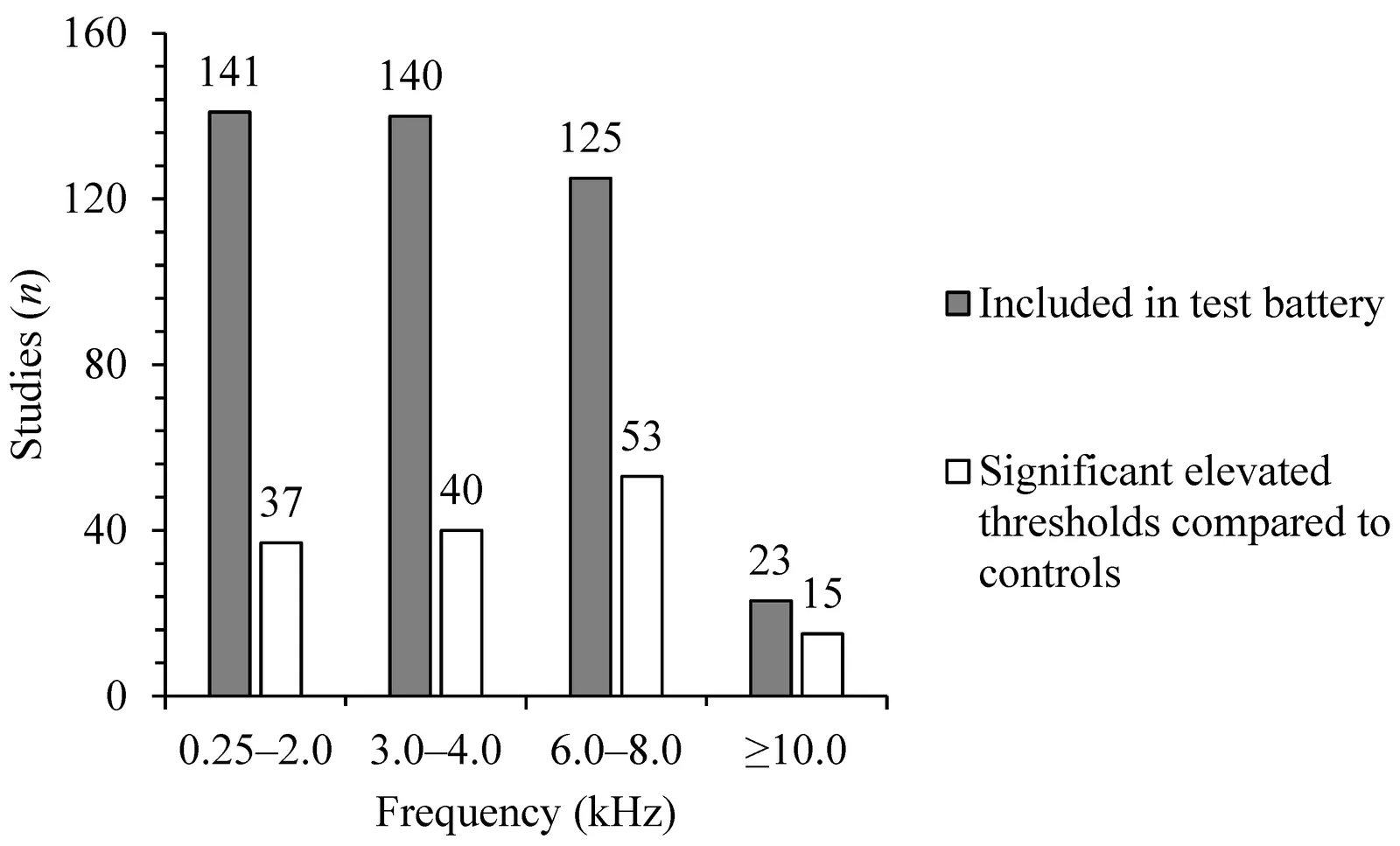
Pure Tone Audiometry Findings.

Although assessment tools were generally well described, the rationale for selecting specific measures was often not reported. Nearly all studies incorporated pure-tone audiometry (PTA), encompassing both standard and extended high frequency assessments. Studies evaluated hearing across low-mid (250–2000 Hz; *n* = 141), mid-high (3000–4000 Hz; *n* = 140), high (6000–8000 Hz; *n* = 125), and extended high frequencies (≥10,000 Hz; *n* = 23), with some reporting significantly elevated thresholds in individuals with DM compared to healthy controls (Figure 2). Data were also extracted on whether air conduction and/or bone conduction testing was performed, and on the reported type of hearing loss. Among studies reporting hearing loss type, 99 reported sensorineural hearing loss only, eight reported sensorineural or conductive hearing loss, and 12 reported sensorineural, conductive, or mixed hearing loss. No studies reported conductive hearing loss as the sole type of hearing loss.

Multiple forms of OAEs and electrophysiological tools were used as diagnostic measures. Types of OAEs included distortion product otoacoustic emissions (DPOAEs; *n* = 32), transient evoked otoacoustic emissions (TEOAEs; *n* = 14), evoked OAEs with the specific type not further specified (*n* = 16), and a single study used stimulus frequency OAEs. Thirteen studies reported reduced OAE amplitude in the group living with DM. In addition, ten of the studies reported absent OAEs in the group living with DM. When considering the electrophysiological measures, most studies included auditory brainstem measures (*n* = 41), with very few including cortical testing (*n* = 2), auditory steady-state response (*n* = 2), or electrocochleography (*n* = 1). Over half of the studies using auditory brainstem responses (ABRs) reported prolonged absolute and/or interwave latencies (*n* = 22). For cortical testing, all studies reported prolonged wave latencies in the group living with DM, and one study reported reduced wave amplitudes in the group living with DM when compared to the control group. Table 1 presents the results from the studies, specific to the different types of OAE and electrophysiological diagnostic measures. Otoscopy (*n* = 32), tympanometry (*n* = 53), acoustic reflex testing (*n* = 29), speech audiometry (*n* = 35), the tuning fork (*n* = 17), and speech-in-noise (SIN) measures (*n* = 4) were also incorporated as an adjunct to standard audiological evaluation or to support inclusion eligibility criteria. 

Results related to SIN testing included a study by Abou-Elew and colleagues [33] that reported a statistically significant difference between individuals with DM and control groups, with individuals living with DM demonstrating poorer performance on the SIN test. Using the QuickSIN, Konrad-Martin and colleagues [34] found that individuals with uncontrolled DM demonstrated poorer SIN performance than the control group, although these differences did not reach statistical significance [35]. Similarly, Falahzadeh and colleagues [36], found that individuals living with DM demonstrated significantly poorer overall SNR-50 thresholds, the signal-to-noise ratio where a person understands 50% of speech, at difficult noise conditions (15, 10, 5, 0 SNR). Iqbal and colleagues [37] found no significant differences in SNR-50 thresholds between individuals living with diabetes for 5–10 years and those with >10 years of disease duration. Correlation analysis indicated a moderately strong association, suggesting that living with DM for a longer duration is associated with greater difficulty listening to speech in noisy conditions [37].

### 3.7. Reported Clinical Recommendations

A larger proportion of studies did not report recommendations (*n* = 118) compared with those that did (*n* = 83). Some recommendations included screening (*n* = 59), hearing check-ups (*n* = 28), follow-up hearing assessments (*n* = 12), preventative care (*n* = 14), risk reduction (*n* = 11), early referral (*n* = 6), collaborative healthcare efforts (*n* = 10), early intervention (*n* = 6), and metabolic assessments (*n* = 2). When recommendations were noted, they varied considerably and were vague. Agrawal and colleagues [38] recommend that patients living with DM undergo periodic audiometric evaluations alongside regular blood glucose monitoring to prevent severe hearing loss, although no specific timeframe for monitoring was provided.

Recommendations regarding assessment approaches also varied across studies. PTA was frequently recommended for screening, likely due to its relatively low cost and widespread availability across referral units and tertiary care centres [39]. In some studies, high frequency PTA was recommended as an essential tool to identify early onset of HL [40,41,42,43,44,45]. Recommendations regarding the timing of PTA varied across studies. Adebola and colleagues [46] suggest PTA assessment at the time of T2DM diagnosis and then at a minimum of six-monthly intervals depending on initial results, while Mishra and Poorey [47] propose annual screening with PTA. One study noted that PTA may not be appropriate for detecting underlying hearing dysfunction in T1DM [48].

Regarding ABR recommendations, one study recommended testing individuals living with DM using ABR once per year, as ABR recordings at higher frequencies, such as 6 kHz and at 80 dB, may facilitate earlier detection of central neuropathy [49]. Three studies also recommend the use of DPOAE for early detection of subclinical auditory dysfunction and as a useful tool to identify individuals who may benefit from regular audiologic monitoring and improved glycemic management [50,51,52].

## 4. Discussion

This scoping review synthesized evidence from 201 peer-reviewed studies examining the association between DM and HL, with a particular focus on approaches to hearing health assessment, monitoring, and management. Although the evidence consistently supports an association between DM and HL, the review identified substantial gaps in clinical practice guidance, including the absence of standardized hearing-monitoring recommendations, inconsistent reporting of referral pathways, and limited integration of audiologists into diabetes care teams. At the same time, the findings provide important direction for future care pathways. The included studies demonstrate that audiological assessments, including behavioural threshold testing, OAEs, and ABRs, each provide complementary information by evaluating hearing sensitivity, cochlear function, and neural auditory function, respectively, and may support the early identification and/or monitoring of diabetes-related auditory changes. The findings also suggest that hearing monitoring strategies could be strengthened by incorporating patient-specific risk factors, such as glycemic control, alongside patient-reported hearing measures to identify individuals who may benefit from earlier or more frequent audiological assessment. Collectively, these findings underscore the need for evidence-informed hearing monitoring recommendations and greater interdisciplinary integration of hearing healthcare into routine diabetes management.

### 4.1. Inconsistent Assessment, Monitoring and Management Strategies

Assessment measures varied significantly across studies, with considerable differences in testing protocols, assessment batteries, and frequency ranges evaluated, limiting comparability across the literature. Recommendations for assessment tools differed across studies, ranging from conventional threshold testing to ABR and OAE testing. As highlighted in the literature, objective measures such as OAEs are well-suited as simple, rapid screening tools [53], while complementary assessments such as PTA provide the audiological information necessary to guide clinical management [54]. Particularly, high and extended high frequency testing is needed, given that high frequency HL is most often observed in individuals living with DM [55]. Similar variability was observed in recommendations for hearing monitoring. While some studies recommended regular auditory assessments, others recommended monitoring at specific intervals, such as annually [47,56]. This variability highlights the need for evidence-based guidance to establish standardized assessment protocols and monitoring schedules for adults living with DM. Future clinical guidelines should balance measurement specificity with clinical feasibility. Although specialized assessments such as high frequency ABR may detect early auditory changes, routine implementation may be constrained by equipment availability, clinician expertise, appointment duration, and healthcare resources. Recommendations should distinguish between essential components that are feasible in routine practice and optional or specialized assessments that can be incorporated when resources permit or clinical indications warrant. 

### 4.2. Subclinical Auditory Dysfunction and Early Detection

Progressive HL often occurs in adults living with DM, with early subclinical changes that can occur before HL becomes detectable using standard audiometric testing. Some of the included studies reported normal hearing thresholds within conventional frequency ranges, despite evidence of auditory dysfunction, including elevated extended high frequency thresholds, reduced OAE amplitudes, or prolonged electrophysiological wave latencies. These findings may suggest that standard behavioural audiometry alone may be insufficient to identify early cochlear and neural changes associated with DM. K.D. Joshi and colleagues [57] noted that although PTA can detect HL, it is limited in its ability to identify early-stage auditory changes and support timely intervention. PTA is less sensitive when it comes to detecting subtle auditory changes due to DM. This limitation may reduce its utility as a standalone screening tool for detecting subtle changes in the auditory system [57]. While incorporating a range of audiological measures may improve early identification and support timely monitoring and intervention, such approaches may not be accessible in all clinical contexts.

### 4.3. Functional Hearing Assessment

In resource-limited settings, initial screening may be conducted with patient-reported outcome measures such as the Hearing Handicap Inventory for the Elderly–Screening Version or a single-question screening approach, with referral for comprehensive audiological evaluation for individuals who report hearing difficulties [58,59]. Four included studies incorporated SIN testing as part of the audiometric assessment battery. Functional hearing extends beyond the measurement of hearing thresholds and reflects an individual’s ability to communicate effectively in everyday listening environments. Assessments may include speech recognition in quiet and noise, patient-reported measures of hearing and communication, ecological or real-world listening assessments, and other measures of auditory function. SIN testing was the only objective functional hearing measure identified in the current literature. Although evidence remains limited, it may be important to incorporate functional hearing assessments alongside conventional audiometric testing to improve the early identification and monitoring of diabetes-related auditory changes and to capture deficits that are not apparent based on PTA alone.

### 4.4. Multi-Level Auditory Involvement in Adult DM

The findings of this review suggest that DM is associated with auditory dysfunction across multiple levels of the auditory system, including cochlear, neural, and functional components. Behavioural assessments such as PTA commonly identify elevated hearing thresholds, most notably at higher frequencies, with poorer hearing outcomes associated with longer disease duration and poorer glycaemic control. Only three studies showed reduced OAEs when both the group living with diabetes and the control group had normal PTA thresholds [60,61,62]. No studies showed a delayed absolute wave V latency when both the group living with diabetes and the control group had normal PTA thresholds. Objective cochlear measures using OAEs indicated early outer hair cell dysfunction, despite normal audiometric thresholds, suggesting subclinical auditory changes. Electrophysiological measures, particularly ABRs, frequently demonstrated delayed wave latencies consistent with slowed neural transmission along the auditory pathway. Although associations between hearing outcomes and clinical factors, such as glycemic control and duration of DM, were not consistently reported across studies, the overall pattern of findings supports a progressive trajectory of hearing loss in people living with DM, characterized by subtle subclinical changes and potential auditory dysfunction at both the cochlear and neural levels.

### 4.5. Age as a Confounding Variable

As individuals age, the prevalence of HL increases, with more than 65% of adults aged 60 years and older experiencing HL [63]. The present scoping review identified 63 studies that included participants living with diabetes older than 60 years of age. Given the high prevalence of age-related HL among older adults, age may represent an important confounding factor when examining the association between DM and HL. Among the included studies involving adults over 60 years of age, only 12 explicitly identified age-related HL as a potential confounder [31,55,57,64,65,66,67,68,69,70,71,72]. Mitchell and colleagues [68] examined the relationship between T2DM and the prevalence, incidence, and progression of age-related HL. Their findings suggest that T2DM may not predispose older individuals to develop age-related HL but may instead exert an additive or interactive effect that contributes to a greater decline in hearing function compared to aging alone. These findings should be interpreted cautiously given the small number of participants living with DM at follow-up in this specific study [68]. More recently, Nisar and colleagues [8] reported that the risk of HL in individuals living with DM was significantly elevated among younger adults, but not among older adults. The lack of significant findings in older adults may reflect the influence of age-related HL masking the effects of diabetes, insufficient age matching between comparison groups, or limited sample size. In contrast, the strong association observed among younger adults, suggests that DM may be a more prominent independent risk factor for HL earlier in adulthood and challenges the assumption that DM-related HL is solely an acceleration of age-related HL. 

Collectively, these findings highlight the importance of considering age when interpreting the relationship between DM and HL. Future research should carefully consider age as a critical factor in study design (e.g., younger versus older adults), analysis, and interpretation of findings to better clarify the independent contribution of DM to hearing outcomes.

### 4.6. Lack of Interdisciplinary Collaboration

DM care is complex, given its effects on multiple body systems, and requires a coordinated interdisciplinary approach and comprehensive management. Although a range of healthcare professionals involved in HL identification were identified across studies, only a subset of studies specified their roles, and these descriptions were often limited. This lack of reporting makes it difficult to determine how hearing care is coordinated for adults living with DM, or whether auditory monitoring is consistently integrated into routine diabetes care. Across the wider literature, studies indicated that awareness of DM-related auditory complications among healthcare professionals remains limited, and referral processes for hearing assessment are often poorly defined or inconsistently applied [73,74]. This may contribute to audiology involvement in DM care occurring primarily after individuals report significant hearing concerns, rather than through routine hearing health monitoring.

Some of the included studies emphasized interdisciplinary collaboration and recommended greater integration among audiologists, endocrinologists, otolaryngologists, primary care physicians, nurses, DM specialists, and health promoters [72,75,76,77,78,79]. Interdisciplinary care models can help to facilitate earlier identification of HL by increasing referrals to audiology services and incorporating hearing screening into routine diabetes management [40,72,76]. Regular hearing evaluations within diabetes care pathways may support earlier detection and management of SNHL, improving overall patient well-being [80]. Mohammed and colleagues [80] suggest that early identification of hearing loss combined with optimal management and treatment of blood glucose and blood pressure may protect against cochlear damage, highlighting the importance of coordinated interdisciplinary care. Public health initiatives also have an important role in raising awareness and providing education regarding the risk of SNHL in individuals with DM and HTN [80]. More broadly, interprofessional care models have been associated with improved clinical outcomes, including reduced complications and enhanced quality of life [81]. Integrating audiology services into established interprofessional care models supported by well-defined clinical pathways and evidence-based guidelines, as part of future research and clinical scenarios, may help promote the comprehensive and patient-centred management of adults living with DM.

### 4.7. Hearing Health as a Shared Responsibility Across Parallel Care Pathways

Building from this scoping review, there is an opportunity to broaden the conceptualization of hearing health within diabetes care, viewing it as both a potential complication of diabetes requiring monitoring and a factor that may influence diabetes management. Hearing difficulties can affect communication with healthcare providers, access to health information, participation in self-management, and engagement in person-centred care [82]. This review also highlights that diabetes-related metabolic and vascular changes may contribute to progressive auditory dysfunction. These reciprocal relationships support the development of parallel, shared person-centred care pathways, in which diabetes care and hearing healthcare function as complementary components of chronic disease management rather than isolated disciplines connected only through referral. Within this model, hearing health becomes a shared responsibility across healthcare providers. Diabetes care teams are well-positioned to raise awareness of the relationship between diabetes and hearing health, identify individuals at increased risk of hearing difficulties, incorporate hearing health discussions into routine care, and facilitate timely referral to audiology. Audiologists, in turn, are well-positioned to contribute expertise in hearing screening, comprehensive assessment, diagnosis, hearing aid fitting, rehabilitation, and, in many jurisdictions, the management of uncomplicated ear conditions [63]. Beyond direct patient care, audiologists also contribute to health promotion, education, advocacy, research, and interdisciplinary care, and are well-suited to identify individuals with unmet chronic disease needs through their work with patients with multiple comorbidities across diverse healthcare and community settings [83].

**Figure 3:**
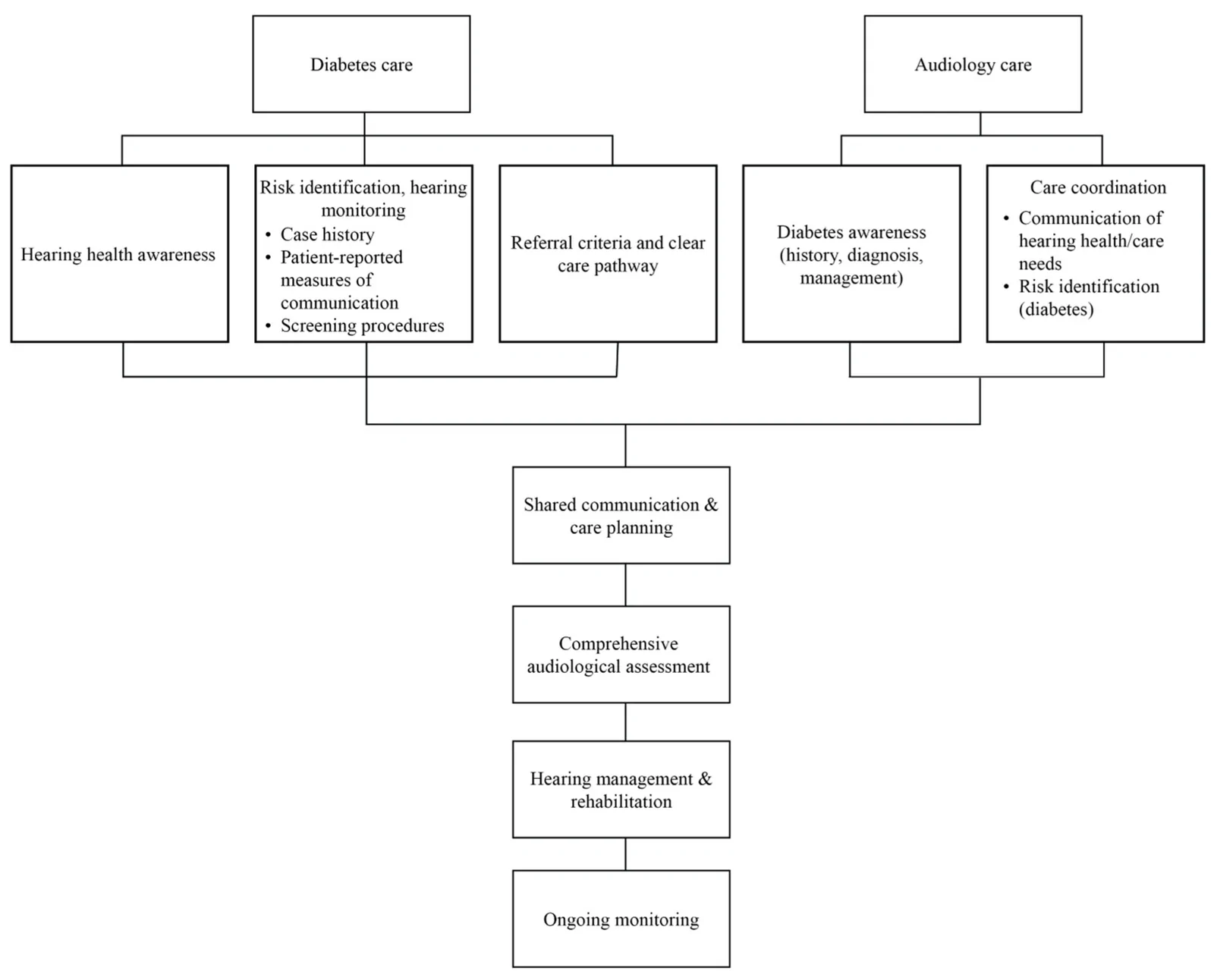
Parallel, Shared Person-Centred Care Pathways in Hearing Loss and Diabetes Management.

Few included studies provided explicit recommendations regarding hearing assessment or monitoring in diabetes, and the available evidence did not establish standardized testing protocols, monitoring intervals, or referral criteria. Accordingly, the framework proposed below should not be interpreted as an evidence-based clinical guideline. Rather, it represents a synthesis of considerations arising from the available literature and identifies areas for future research, consensus development, and clinical guidance. A stepped hearing healthcare pathway embedded within these parallel care pathways has been proposed and may provide a practical framework for integrating hearing health into routine diabetes management (Figure 3). The pathway would begin with hearing health education for all individuals living with diabetes to increase awareness of the relationship between diabetes and hearing health, encourage self-monitoring, and promote discussion of hearing concerns during routine diabetes encounters. Individuals with established risk factors, such as longer disease duration, suboptimal glycemic control, increasing age, or self-reported communication difficulties, could then undergo targeted hearing health monitoring using standardized case-history questions, patient-reported outcome measures (e.g., subjective and objective measures of hearing function), and feasible hearing screening procedures (e.g., OAEs). Those with identified hearing concerns or abnormal screening findings would be referred for comprehensive audiological assessment and individualized management, with ongoing bidirectional communication between audiologists and diabetes care providers to support coordinated, longitudinal, person-centred care.

Although the optimal monitoring intervals, referral criteria, and assessment protocols remain to be established, future clinical guidelines should balance scientific evidence with clinical feasibility. Standardized care pathways incorporating evidence-informed risk stratification, monitoring schedules, referral thresholds, and assessment recommendations could promote more consistent and proactive hearing healthcare. In diabetes management, stepped care pathways will be needed to distinguish between core monitoring components that are feasible across most practice settings and enhanced assessments that may be reserved for higher-risk individuals or implemented when resources permit. This stepped approach has the potential to improve implementation while maintaining opportunities for more comprehensive evaluation when clinically indicated. Importantly, hearing loss is not only a potential complication of diabetes but may also influence chronic disease management by reducing the quality of patient-provider communication, thereby limiting opportunities for education, shared decision-making, and the timely identification of other health concerns [82,83]. Integrating hearing health within routine diabetes care may therefore facilitate earlier identification of hearing loss, improve access to appropriate interventions, support communication and participation, and ultimately enhance quality of life for individuals living with diabetes [8]. Future research should aim to establish and evaluate person-centred hearing healthcare pathways across diverse diabetes care settings.

## 5. Limitations and Future Directions

Several limitations should be considered when integrating the findings of this review. The search was restricted to English-language, peer-reviewed publications, potentially excluding relevant studies. Most of the included studies employed cross sectional or case–control designs, with only six longitudinal follow-up studies identified. This limits the ability to understand the progression of HL over time and the long-term impact of DM on hearing health across the lifespan. The lack of randomized controlled trials limits the strength of conclusions about the effectiveness of interventions to prevent or manage HL in adults with DM. Future research should prioritize longitudinal and experimental study designs and improve consistency in reporting key clinical factors, such as age at DM onset and diabetes duration, to strengthen the evidence base and guide effective management strategies. The reporting of care pathways and health professional involvement was inconsistent across studies, limiting understanding of how HL identification and management are currently coordinated within DM care. More comprehensive reporting of healthcare provider roles and interdisciplinary collaboration is required to facilitate the integration of audiology into DM care. Finally, none of the included studies addressed the role of caregivers in supporting adults living with DM and HL. Although caregivers may play an important role in supporting diabetes self-management and hearing healthcare outcomes, caregiver needs and supports remain underexplored [84]. Future research should consider caregiver involvement, including education and family counselling strategies, as part of a comprehensive management approach for this population.

## 6. Conclusions

This scoping review synthesizes the existing evidence on the early detection, assessment, and monitoring of hearing loss among adults living with DM, with consideration of the role of care pathways in supporting the identification and management processes. The findings suggest inconsistencies in recommended diagnostic methods, limited description or reporting of referral pathways to audiology services, and a lack of attention to interdisciplinary collaboration within integrated models of care. Furthermore, the observed variability in how hearing assessments are incorporated into standard DM management underscores the lack of standardized protocols for identifying and addressing auditory complications in this population. Establishing structured, evidence-based guidelines for hearing screening and monitoring among adults living with DM may support earlier identification of auditory changes and facilitate timely intervention leading to improved communication, greater social participation and overall well-being. Similarly, clearer articulation of referral pathways and defined roles for healthcare providers may strengthen coordination across disciplines and support more integrated models of care. Greater integration of audiology services within interdisciplinary DM care teams also presents an important opportunity to enhance the continuity and comprehensiveness of care. Incorporating routine hearing assessments into established DM management pathways, supported by increased clinician awareness and engagement with family and caregivers, may promote more proactive identification and management of hearing health.

Future research should prioritize the development and evaluation of implementation strategies, including longitudinal and intervention-based study designs, to inform effective evidence-based models for integrating hearing health into DM care. Collectively, the findings of this review support a shift toward earlier, more comprehensive, and collaboratively delivered models of hearing health monitoring and management for adults living with DM. This shift will require moving beyond reactive approaches that address hearing concerns only after they emerge, toward proactive identification of HL, standardized monitoring pathways, and greater integration of audiology within interdisciplinary diabetes management.

## Figures and Tables

**Table 1:** Otoacoustic Emissions (OAEs) and Electrophysiology Measurements.

Test Type	Studies (*n*)	Findings Related to HL	Additional Findings
OAE measurements			
SFOAE	1	SFOAE latency and amplitude results were not reported.	
TEOAE	14	Reduced TEOAE amplitude in the group living with DM compared to the control group was reported in *six* studies. Absent TEOAEs in the group living with DM were found in *three* studies.	One study reported no effect of duration and HbA1c levels on TEOAE amplitude [25]. The mean amplitude of TEOAE showed a negative linear correlation with age [26].
DPOAE	32	Reduced DPOAE amplitude in the group living with DM compared to the control group was reported in *ten* studies. Absent DPOAEs in the group living with DM were found in *six* studies.	One study reported no effect of duration and HbA1c levels on DPOAE amplitude [25]. One study reported no effect of duration on latency of the DPOAE [27]. Four studies reported that higher HbA1c levels were associated with reduced DPOAE amplitudes or absent DPOAE responses [28,29,30,31]. The absence of DPOAEs occurred mostly in adults older than 50+ years [31].
Electrophysiological measurements			
ABR	41	Absolute/interwave latency prolongations were reported in over half of the studies (*n* = 22). Waveform morphology appeared to fall within expectations in studies that assessed morphology.	No consistent finding was evident correlating the ABR results to duration of the disease, age of disease onset or blood glucose levels.
Cortical Response	2	Absolute latencies for waves P1, N1, and P2 were prolonged in the group living with DM.	No correlation with glycated hemoglobin was evident. There was a significant positive correlation observed for latency and duration of T2DM [32].

## Data Availability

No new data were created or analyzed in this study.

## Supplementary Materials

The following supporting information can be downloaded at: https://www.mdpi.com/article/10.3390/audiolres16050134/s1, File S1: Data Charting Tool, File S2: Preferred Reporting Items for Systematic Reviews and Meta-Analyses extension for Scoping Reviews (PRISMA-ScR) Checklist.

## Abbreviations

ABR	Auditory brainstem response
BMI	Body mass index
CAEP	Cortical auditory evoked potentials
DM	Diabetes mellitus
DPOAE	Distortion product otoacoustic emissions
EOAE	Evoked otoacoustic emission
HbA1c	Hemoglobin A1c
HL	Hearing loss
HTN	Hypertension
IDDM	Insulin-dependent diabetes mellitus
JBI	Joanna Briggs Institute
NIDDM	Non-insulin-dependent diabetes mellitus
OAE	Otoacoustic emission
PRISMA-ScR	Preferred Reporting Items for Systematic reviews and Meta-Analyses extension for Scoping Reviews
PTA	Pure-tone audiometry
SFOAE	Stimulus frequency otoacoustic emissions
SIN	Speech-in-noise
SNHL	Sensorineural hearing loss
SNR	Signal-to-noise ratio
SNR-50	Signal-to-noise ratio-50
SPOAE	Spontaneous evoked otoacoustic emissions
SSNHL	Sudden sensorineural hearing loss
T1DM	Type 1 diabetes mellitus
T2DM	Type 2 diabetes mellitus
TEOAE	Transient evoked otoacoustic emissions
WHO	World Health Organization

## Appendix A. Search Strategy

Ovid MEDLINE Search Strategy hearing disorder*.ti,ab.

1. diabet*.ti,ab. deaf*.ti,ab.
2. hearing loss.ti,ab. auditory impair*.ti,ab.
3. hearing impair*.ti,ab. hearing defic*.ti,ab.
4. hearing disab*.ti,ab. hearing difficult*.ti,ab.
5. partial deaf*.ti,ab. hearing decline*.ti,ab.
6. hearing reduc*.ti,ab. loss of hearing.ti,ab.
7. hearing challenge*.ti,ab. hearing dysfunction*.ti,ab.
8. auditory loss.ti,ab.
9. normal hearing.ti,ab.
10. functional hearing.ti,ab.
11. typical hearing.ti,ab.
12. 2 or 3 or 4 or 5 or 6 or 7 or 8 or 9 or 10 or 11 or 12 or 13 or 14 or 15 or 16 or 17 or 18 or 19
13. 1 and 20

## Appendix B

**Table A1:** Summary of Study Characteristics, Assessment Methods, and Clinical Recommendations.

Citation	Research Design	CEBM ^1^ Level	Mean Age & Range of Group Living with DM (Years)	Comorbidities Tested	Healthcare Professionals Involved in Audiological Care	Audiological Tools for Screening & HL Identification ^2^	Recommendations ^3^
Abida et al. [86]	Cross sectional	3	Range: ≥30–>70	HTN (hypertension)	Unspecified	PTA (pure tone audiometry)	Screening
AbidAlkadem et al. [87]	Cross sectional	3	Range: 25–75	Tinnitus; headache; otalgia	Unspecified	PTA; otoscopy; HFA (high-frequency audiometry)	Follow-up hearing assessment
Abou-Elew et al. [33]	Case–control	4	Mean: 51.23 Range: <60	Mild and moderate cognitive impairment	Unspecified	PTA; speech audiometry; tympanometry; SIN (speech in noise); acoustic reflex testing	Hearing check-up
Adebola et al. [46]	Cross sectional	3	Mean: 58.09 Range: 30–≥80	Overweight; obesity; HTN	Audiologist; otolaryngologist	PTA; otoscopy	Screening
A.C. Agarwal et al. [88]	Cross sectional	3	Range: 18–50	None provided	Audiologist	PTA; tympanometry; TEOAE (transient evoked otoacoustic emissions); EOAE (evoked otoacoustic emissions)	Early referral
M.K. Agarwal et al. [89]	Cross sectional	3	Mean: 50.76 Range: 20–80	None provided	Unspecified	PTA	None
Agrawal et al. [38]	Cross sectional	3	Range: 25–75	None provided	Unspecified	PTA	Hearing check-ups
Akbar et al. [90]	Cross sectional	3	Range: 30–60	None provided	Unspecified	PTA	Screening; monitor hearing
Akbar & Kamalakshi [91]	Cross sectional	3	Mean: 50.86 Range: 30–60	None provided	Unspecified	PTA	Hearing check-ups
Al-Abed et al. [92]	Cross sectional	3	Mean: 60.57	HTN; retinopathy; tinnitus	Audiologist	PTA	None
Al-Rubeaan et al. [28]	Cross sectional	3	Mean: 51 Range: 47–54	Neuropathy; retinopathy; nephropathy; vasculopathy	Unspecified	PTA; tympanometry; DPOAE	Screening; risk reduction
Al-Sofiani et al. [93]	Cross sectional	3	Mean: 43.8 Range: 20–60	Retinopathy; nephropathy; neuropathy; chronic artery disease; stroke/TIA; peripheral artery disease	Unspecified	PTA	None
Aladag et al. [94]	Cross sectional	3	Mean: 46.58	None provided	Unspecified	PTA; tympanometry; otoscopy	None
Alenzi et al. [95]	Cross sectional	3	Mean: 49.1	None provided	Unspecified	PTA	Follow-up hearing assessment; screening; hearing check-ups; preventative care
Alizadeh et al. [96]	Case–control	3	Mean: 59.8	HTN; dyslipidemia; retinopathy	Unspecified	PTA	Screening
AlJasser et al. [48]	Cross sectional	3	Mean: 26.8 Range: 19–35	Neuropathy; retinopathy	Unspecified	PTA; ABR	Screening
Allayla et al. [97]	Case–control	3	Mean: 33 Range: 18–45	None provided	Audiologist technician	PTA; tympanometry; acoustic reflex testing; otoscopy	None
Allayla et al. [98]	Case–control	3	Mean: 33 Range: 18–45	None provided	Unspecified	PTA; ABR	Audiological evaluation
Aremu et al. [99]	Cross sectional	3	Mean: 65.3 Range: 43–82	None provided	Audiologist	PTA; DPOAE	Screening
Asghar et al. [75]	Cross sectional	3	Mean: 48.61 Range: 18–60	Vision problem; neuropathy; ischaemic heart disease; HTN	Audiologist	PTA	Risk reduction; collaborative healthcare efforts
Ashkezari et al. [100]	Cross sectional	3	Mean: 56.38 Range: 40–65	Cardiovascular factors; nephropathy; retinopathy	Audiologist	PTA	None
Austin et al. [101]	Cross sectional	3	Range: 26–71	Retinopathy; neuropathy; nephropathy	Audiologist	PTA; SFOAE (stimulus frequency otoacoustic emission)	None
Babu et al. [102]	Cross sectional	3	Range: 31–60	None provided	Unspecified	PTA; otoscopy	Follow-up hearing assessment; screening
Bainbridge et al. [103]	Cross sectional	1	Range: 18–76	HTN; chronic kidney disease; heart disease; cerebrovascular disease; cholesterol; intermittent claudication (symptom of peripheral artery disease)	Technician (not specified)	PTA	None
Bainbridge et al. [104]	Cross sectional	1	Range: 20–69	None provided	Audiometric technician	PTA; otoscopy	Screening
Bainbridge et al. [105]	Cross sectional	1	Range: 40–69	HTN; neuropathy; cardiovascular risk factors (e.g., obesity, high-density lipoprotein, HTN, and smoking)	Audiometric technician	PTA	None
Bamanie & Al-Noury [106]	Case–control	3	Mean: 47.29 Range: 29–69	Neuropathy; hyperlipidemia; cataract; HTN; retinopathy; heart disease; renal disease; diabetic foot	Unspecified	PTA	Counselling
Barreto et al. [107]	Cross sectional	3	Range: 31–60	None provided	Unspecified	PTA	Early referral
Batni et al. [108]	Cross sectional	3	Mean: 41.9 Range: 40–80	None provided	Unspecified	PTA; speech audiometry; otoscopy	None
Bener et al. [109]	Cross sectional	3	Unspecified	HTN; smoking; obesity; hypoglycemia; retinopathy; nephropathy; neuropathy; macro-vascular disease; tinnitus; vertigo	Unspecified	PTA	Screening
Bener et al. [110]	Cross sectional	3	Range: 50+	Retinopathy; nephropathy; neuropathy; tinnitus; vertigo	Technician (not specified)	PTA	None
Bodat [111]	Observational	3	Range: 30–70	None provided	Unspecified	PTA; tympanometry; otoscopy	Screening
Braffett et al. [19]	Cohort	3	Mean: 55	HTN; retinopathy; neuropathy; cardiovascular disease; coronary artery calcification	Audiologist	PTA	None
Çayönü et al. [112]	Cross sectional	3	Mean: 71.5 Range: 65–89	None provided	Unspecified	PTA; tympanometry	None
Celik et al. [113]	Cross sectional	3	Mean: 45.3 Range: 14–60 *	Neuropathy; retinopathy; nephropathy; vascular disease	Unspecified	PTA	None
Chamyal [114]	Cross sectional	3	Range: 17–49 *	None provided	Unspecified	PTA; tuning fork	None
Chaudhari & Mehta [115]	Cross sectional	3	Unspecified	None provided	Unspecified	PTA	None
Chavan et al. [78]	Cross sectional	3	Mean: 46.06 Range: 30–60	None provided	Unspecified	PTA	Screening; hearing check-ups; counselling; preventative care; collaborative healthcare efforts
Chee et al. [116]	Cross sectional	3	Range: 60–100	HTN; hyperlipidaemia	Audiologist	PTA	Screening
Cheng et al. [117]	Cross sectional	1	Mean: 44.9 Range: 25–69	None provided	Unspecified	PTA	None
Cho et al. [50]	Cross sectional	3	Mean: 55.1 Range: 41–76	None provided	Unspecified	PTA; speech audiometry; ABR; DPOAE	Screening
Cullen & Cinnamond [118]	Cross sectional	3	Mean: 46.9	None provided	Unspecified	PTA; speech audiometry; acoustic reflex testing	None
Dabrowski et al. [26]	Cross sectional	3	Mean: 29.1 Range: 18–43	Retinopathy	Unspecified	PTA; ABR; TEOAE; EOAE	Hearing check-ups
Dadhich et al. [119]	Prospective	3	Range: 31–59	None provided	Unspecified	PTA	None
Dalton et al. [64]	Longitudinal	3	Mean: 69.6 Range: 43–84	HTN; smoking; retinopathy; nephropathy	Audiologist; technician (not specified)	PTA; tympanometry	None
Das et al. [55]	Cross sectional	3	Mean: 43.63 Range: 21–64	Overweight	Unspecified	PTA; HFA	None
de Almeida Beretta et al. [120]	Retrospective	3	Mean: 35 Range: 19–59	None provided	Unspecified	PTA	None
De Espana et al. [121]	Cross sectional	3	Range: 7–47 *	None provided	Unspecified	PTA; ABR; HFA	None
De Leon-Morales et al. [122]	Cross sectional	3	Mean: 50	Neuropathy; retinopathy; nephropathy; microalbuminuria	Unspecified	PTA; speech audiometry; ABR	None
Dhasmana et al. [123]	Cross sectional	3	Mean: 47.18 Range: 18–59	None provided	Unspecified	PTA; tuning fork	Screening
Dillard et al. [124]	Longitudinal	3	Mean: 68.5	None provided	Unspecified	PTA; speech audiometry; ABR; EOAOE; otoscopy;	None
Di Nardo et al. [25]	Cross sectional	4	Unspecified	Peripheral neuropathy	Unspecified	PTA; TEOAE; DPOAE; EOAE	None
Diniz & Guida [125]	Cross sectional	3	Mean: 65.5 Range: 45–83	Dysacusis; HTN; tinnitus	Unspecified	PTA; speech audiometry; tympanometry; acoustic reflex testing	Follow-up hearing assessment
Donald et al. [126]	Cross sectional	3	Mean: 38 Range: 20–60	None provided	Unspecified	PTA; ABR; speech audiometry; tympanometry; acoustic reflex testing; otoscopy	None
Dosemane et al. [23]	Cross sectional	3	Range: 40–60	None provided	Trained personnel (not specified)	PTA; ABR	Hearing check-ups
El-Tabal et al. [127]	Cross sectional	3	Range: <60	Retinopathy; nephropathy; ischaemic heart disease; peripheral vascular disease; neuropathy	Unspecified	PTA	None
Elibol & Baran [56]	Cross sectional	3	Mean: 39.82 Range: 18–65	Microangiopathy	Unspecified	PTA; speech audiometry	Follow-up hearing assessment
ElSherif et al. [128]	Case–control	3	Mean: 50.67 Range: 39–60	None provided	Unspecified	PTA; speech audiometry; tympanometry; acoustic reflex testing; otoscopy; high-frequency audiometry	Hearing check-ups; metabolic assessment
Erdem et al. [129]	Cross sectional	3	Mean: 48.6 Range: 33–57	Hyperlipoproteinemia; hypertriglyceridemia	Unspecified	PTA; tympanometry; TEOAE; DPOAE; EOAE	None
Eren et al. [130]	Cross sectional	3	Mean: 47.6	None provided	Unspecified	PTA; tympanometry; acoustic reflex testing; TEOAE; DPOAE; EOAE	None
Esubalew et al. [20]	Cross sectional	1	Mean: 51.6 Range: 24–70	HTN; hyperlipidemia; thyroid; cardiovascular disease	Audiologists	PTA	Screening; risk reduction
Falahzadeh et al. [36]	Cross sectional	3	Mean: 46.17 Range: 30–60	None provided	Unspecified	PTA; speech audiometry; tympanometry; SIN	None
Fathima et al. [131]	Cross sectional	3	Range: 35–70	None provided	Unspecified	PTA; tuning fork	Screening
Felicio et al. [51]	Cross sectional	3	Mean: 23	Diabetic kidney disease; diabetic cardiac autonomic neuropathy	Unspecified	PTA; tympanometry; acoustic reflex testing; DPOAE; otoscopy	Screening
Fernandes et al. [132]	Cross sectional	3	Mean: 21	Hyperglycemia; hypoglycemia	Unspecified	PTA; tympanometry; ABR; acoustic reflex testing; otoscopy	Early intervention; hearing check-ups
Friedman et al. [133]	Cross sectional	3	Mean: 52 Range: 22–70	Retinopathy; peripheral arterial disease; nephropathy	Unspecified	PTA	None
Frisina et al. [65]	Cross sectional	3	Mean: 73 Range: 59–92	None provided	Unspecified	PTA; speech audiometry; tympanometry; TEOAE; DPOAE; EOAE	None
Gawron et al. [134]	Case report	5	Mean: 19	None provided	Unspecified	PTA; tympanometry; ABR; DPOAE	None
Ghosh et al. [135]	Case–control	3	Mean: 46.78 Range: <60	None provided	Unspecified	PTA	Screening
Giraudet et al. [31]	Prospective	3	Mean: 60.4 Range: 23–79	Nephropathy; neuropathy; retinopathy, ischaemic heart disease	Unspecified	PTA; tympanometry; acoustic reflex testing; DPOAE	Screening
Gündoğan et al. [136]	Case–control	3	Mean: 50 Range: 40–60	Retinopathy	Unspecified	PTA; speech audiometry; tympanometry; high-frequency audiometry	Screening; preventative care
Gupta et al. [137]	Case–control	3	Mean: 49.96 Range: 40–60	None provided	Unspecified	PTA; tympanometry; ABR; acoustic reflex testing	None
Gupta et al. [138]	Cross sectional	3	Mean: 45.7 Range: 35–50	None provided	Unspecified	ABR; tuning fork	None
Gutierrez et al. [139]	Cross sectional	3	Mean: 57.52 Range: ≥19	HTN; nephropathy; retinopathy; neuropathy; smoking	Unspecified	PTA	Screening; risk reduction
He et al. [140]	Cross sectional	3	Unspecified	HTN; dyslipidemia; overweight, obesity; smoking	Unspecified	PTA	None
Hilali et al. [60]	Cross sectional	3	Mean: 31.6 Range: 23–42	Microangiopathy; neuropathy	Otologist; diabetologist	PTA; tympanometry; acoustic reflex testing; TEOAE; EOAE	None
Hlayisi et al. [40]	Cross sectional	3	Range: 18–55	HTN; retinopathy; neuropathy	Unspecified	PTA; tympanometry; DPOAE	Screening; collaborative healthcare efforts; early referral
Horvath et al. [141]	Prospective	3	Mean: 38.05 Range: 19–60	Chronic kidney disease; retinopathy; neuropathy; hyperlipidemia	Unspecified	PTA; tympanometry; ABR; DPOAE	None
Hu et al. [142]	Cross sectional	3	Mean: 53.8 Range: 30–65	HTN; retinopathy; nephropathy	Unspecified	PTA; tympanometry; TEOAE; EOAE	None
H. Huang et al. [66]	Cross sectional	1	Mean: 53.1 Range: 20–87	HTN; dyslipidemia	Trained personnel (not specified)	PTA; otoscopy	Risk reduction
Y.M. Huang et al. [143]	Cross sectional	3	Mean: 42.4 Range: 14–62 *	Retinopathy; neuropathy; nephropathy	Unspecified	PTA; ABR; speech audiometry; acoustic reflex testing; CAEP (cortical auditory evoked potentials); electrocochleography	None
Iqbal et al. [37]	Cross sectional	3	Mean: 48.11 Range: 30–65	None provided	Unspecified	PTA; speech audiometry; tympanometry; SIN;	Hearing check-ups
Irshad et al. [144]	Case–control	3	Mean: 54.87 Range: 15–60 *	HTN; retinopathy; peripheral neuropathy	Researcher under consultant supervision	PTA	Screening
Jabeen & Narayanan [45]	Prospective	3	Range: 35–65	HTN	Unspecified	PTA; otoscopy; high-frequency audiometry	Screening; hearing check-ups; follow-up hearing assessment; early intervention; risk reduction
K.D. Joshi et al. [57]	Cross sectional	3	Mean: 50.47 Range: 19–79	None provided	Unspecified	PTA; otoscopy	Screening; follow-up hearing assessment
S.S. Joshi et al. [145]	Case–control	3	Mean: 49.64 Range: <18	None provided	Unspecified	PTA	Screening; hearing check-ups
Kabeya et al. [146]	Cross sectional	3	Mean: 64.1	HTN; hypercholesterol; hypertriglyceridemia; obesity	Trained personnel (not specified)	PTA	None
Kakarlapudi et al. [147]	Retrospective	3	Unspecified	Triglycerides	Unspecified	PTA; speech audiometry	None
Kalra [148]	Cross sectional	3	Mean: 57.4 Range: 35–74	HTN	Unspecified	PTA; speech audiometry; tympanometry	None
Khan et al. [149]	Cross sectional	3	Range: 30–>70	HTN	Unspecified	PTA; high-frequency audiometry	Screening; hearing assistive technology; hearing check-ups; counselling; follow-up hearing assessment; preventative care; risk reduction
Kiakojouri et al. [150]	Cross sectional	3	Mean: 50.1 Range: 45–55	None provided	Unspecified	PTA; speech audiometry	None
D.Y. Kim et al. [151]	Longitudinal	3	Unspecified	HTN; cholesterol; smoking; alcohol	Audiometric technician	PTA; otoscopy	None
M.-B. Kim [152]	Prospective	3	Mean: 37.6 Range: 18–87.1	HTN; smoking; alcohol	Audiometric technician	PTA	Hearing check-up
Kishnani et al. [153]	Cross sectional	3	Range: 30–75	None provided	Unspecified	PTA	Screening
Konrad-Martin et al. [154]	Cross sectional	3	Range: 26–71	HTN; foot neuropathy; urinary microalbumin; retinal findings	Unspecified	PTA; SIN; ABR; HFA	None
Konrad-Martin et al. [67]	Cross sectional	3	Mean: 49 Range: 30–66	HTN; heart disease; stroke	Unspecified	PTA; tympanometry; ABR; acoustic reflex testing; HFA; CAEP	None
Konrad-Martin et al. [34]	Longitudinal	3	Mean: 49.4	Retinopathy; peripheral neuropathy; smoking	Audiologist	PTA; speech audiometry; HFA	None
Krishnappa & Naseeruddin [21]	Cross sectional	3	Range: 50+	Neuropathy; angiopathy	Unspecified	PTA	None
Kumar et al. [32]	Cross sectional	3	Mean: 52.16 Range: 40–60	None provided	Unspecified	PTA; speech audiometry; tympanometry; acoustic reflex testing; CAEP	None
Kumar & Purushotham [155]	Retrospective	3	Mean: 45 Range: 40–60	None provided	Unspecified	PTA	Screening
Kumari et al. [79]	Cross sectional	3	Range: 18–55	None provided	Unspecified	PTA	Hearing check-ups; collaborative healthcare efforts
Kurien et al. [156]	Cross sectional	3	Range: <50 *	HTN; retinopathy; neuropathy; nephropathy; vascular disease	Unspecified	PTA	None
Kurt et al. [157]	Cross sectional	3	Mean: 58.3	HTN; chronic kidney disease; retinopathy; neuropathy; cardiovascular disease; vascular disease; microalbuminuria	Unspecified	PTA	None
Lerman-Garber et al. [158]	Cross sectional	3	Mean: 42 Range: 30–50	HTN; retinopathy; tinnitus; cholesterol; peripheral neuropathy; rheumatoid arthritis	Unspecified	PTA; speech audiometry; tympanometry; ABR; acoustic reflex testing; DPOAE; HFA	Screening
J. Li et al. [24]	Cross sectional	3	Mean: 56.1 Range: 20–70	HTN; retinopathy; peripheral neuropathy; tinnitus; diabetic foot ulceration	Unspecified	PTA; DPOAE	Screening
Y. Li et al. [159]	Cross sectional	3	Mean: 45.8 Range: ≤60	Retinopathy; peripheral neuropathy; nephropathy	Unspecified	PTA; tympanometry; DPOAE; HFA	None
Lisowska et al. [61]	Cross sectional	3	Mean: 33 Range: 21–42	Microangiopathy	Unspecified	PTA; ABR; acoustic reflex testing; DPOAE	None
Madhan et al. [160]	Cross sectional	3	Mean: 44.87 Range: 35–55	Dyslipidemia	Unspecified	PTA	None
Mahallik et al. [161]	Cross sectional	3	Mean: 36.64 Range: 25–45	None provided	Unspecified	PTA; ABR; tuning fork	None
Malucelli et al. [41]	Cross sectional	3	Mean: 25.9 Range: 18–55	None provided	Unspecified	PTA; speech audiometry; HFA	Screening
Marquardt & Loriaux [162]	Case report	3	Range: 16–19 *	Retinopathy; neurologic problems; thyroid problem; endocrine abnormalities	Unspecified	PTA	None
Mendonca et al. [163]	Cross sectional	3	Mean: 53 Range: <40–>60	Chronic kidney disease; retinopathy; neuropathy	Unspecified	PTA	Screening
Meneses-Barriviera et al. [164]	Cross sectional	3	Range: 60–90	HTN; tinnitus	Unspecified	PTA; otoscopy	None
Miller et al. [165]	Cross sectional	3	Mean: 43 Range: 22–72	Retinopathy; tinnitus; inactive chronic otitis media	Unspecified	PTA	None
Mishra & Poorey [47]	Cross sectional	3	Range: 50+	None provided	Unspecified	PTA; speech audiometry	Screening; hearing check-ups
I.S. Mishra et al. [49]	Cross sectional	4	Mean: 38.16 Range: 35–50	None provided	Unspecified	PTA; ABR; otoscopy; tuning fork	Hearing check-ups
R. Mishra et al. [166]	Cross sectional	3	Mean: 37.3 Range: 30–40	None provided	Unspecified	PTA; tympanometry; acoustic reflex testing	Screening
U.P. Mishra et al. [167]	Cross sectional	3	Mean: 49 Range: 44–58	HTN; chronic kidney disease; retinopathy; neuropathy	Unspecified	PTA; tympanometry; TEOAE; DPOAE; EOAE	Follow-up hearing assessment; screening
Misra et al. [168]	Cross sectional	3	Range: 30–50+	None provided	Unspecified	PTA; ABR; tympanometry; acoustic reflex testing	None
Misra et al. [169]	Case report	3	Mean: 55	None provided	Otolaryngologist	Tuning fork	None
Mitchell et al. [68]	Longitudinal	1	Mean: 70.5 Range: 55–80+	HTN	Audiologist	PTA	Early referral; preventative care
Mittal et al. [170]	Cross sectional	3	Mean: 59.1 Range: 30–70	Retinopathy; neuropathy	Unspecified	ABR	None
Mohammed et al. [80]	Cross sectional	3	Unspecified	HTN	Unspecified	PTA; otoscopy	Screening; hearing check-up; early intervention; collaborative healthcare efforts
Mohapatra et al. [39]	Cross sectional	3	Range: 20–50	None provided	Unspecified	PTA	Hearing check-up; screening
Moon et al. [171]	Cross sectional	1	Mean: 48.8 Range: 47.0–50.6	HTN; dyslipidemia; smoking	Unspecified	PTA	None
Morrison et al. [172]	Retrospective	3	Mean: 75.3 Range: 41.3–96.7	Retinopathy; chronic kidney disease; peripheral neuropathy; cardiovascular disease; micro/macroalbuminuria; foot ulceration	Unspecified	PTA; tympanometry; otoscopy	Preventative care
Mozaffari et al. [173]	Cross sectional	3	Mean: 45 Range: 21–59	Retinopathy; nephropathy; neuropathy; cerebrovascular disease; cardiovascular disease	Otolaryngologist	PTA	Screening; metabolic assessment; hearing check-up
Munjal et al. [29]	Prospective	3	Range: <30–>50	None provided	Unspecified	PTA; DPOAE	None
Nagahama et al. [174]	Longitudinal	3	Mean: 45 Range: 30–65	HTN; dyslipidaemia; alcohol consumption	Trained personnel (not specified)	PTA; HFA	None
Naik & Tilloo [175]	Cross sectional	3	Range: 30–60	Vestibulopathy	Unspecified	PTA	Screening; risk reduction
Nemati et al. [42]	Cross sectional	3	Mean: 53.74 Range: 39–60	Diabetic retinopathy; nephropathy; diabetic foot	Unspecified	PTA; speech audiometry; tympanometry; acoustic reflex testing; DPOAE; HFA	Follow-up hearing assessment; screening
Nkosi et al. [76]	Cross sectional	3	Mean: 41.03 Range: 21–55	HTN	Unspecified	PTA; ABR; speech audiometry; tympanometry; DPOAE; HFA	Hearing check-ups; counselling; collaborative healthcare efforts
Nwosu & Chime [176]	Case–control	3	Unspecified	None provided	Unspecified	PTA	Hearing check-ups; screening
Ogundiran et al. [71]	Retrospective	3	Mean: 73 Range: ≥65	HTN	Unspecified	PTA	Screening; hearing check-ups; risk reduction
Oh et al. [177]	Cross sectional	3	Mean: 44.1	HTN	Unspecified	PTA	Counselling
Ologe et al. [178]	Cross sectional	3	Mean: 56.95 ± 8.86	None provided	Unspecified	PTA	None
Osterhammel & Christau [179]	Cross sectional	4	Range: 20–49	Retinopathy; nephropathy; neuropathy	Unspecified	PTA; speech audiometry; tympanometry; acoustic reflex testing; HFA	None
Özel et al. [180]	Cross sectional	3	Mean: 50.3 Range: 23–60	None provided	Unspecified	PTA; speech audiometry; tympanometry; acoustic reflex testing	None
Ozkurt et al. [181]	Case–control	3	Mean: 45.9 Range: 40–50	None provided	Unspecified	PTA; HFA	Follow-up hearing assessment; screening
Paluru et al. [182]	Case–control	3	Range: 30–55	None provided	Unspecified	DPOAE	None
Park et al. [27]	Cross sectional	3	Range: 40–63	Retinopathy	Unspecified	PTA; tympanometry; DPOAE	None
Parmar et al. [183]	Cross sectional	3	Mean: 57.3	Hyperlipidemia; high low-density lipoprotein; low high-density lipoprotein	Unspecified	PTA	None
Parving et al. [184]	Cross sectional	3	Unspecified	Peripheral neuropathy; retinopathy; nephropathy; microangiopathy	Unspecified	PTA; ABR; tympanometry; acoustic reflex testing; otoscopy	None
Patel et al. [185]	Prospective	3	Mean: 52.6 Range: 18–70	Retinopathy; neuropathy	Unspecified	PTA	Screening; hearing check-ups; risk reduction; early intervention; collaborative healthcare efforts; preventative care
D. Patil et al. [186]	Observational	3	Mean: 51.76 Range: >18	HTN	Unspecified	PTA	Screening; hearing check-ups; early intervention
S. Patil et al. [77]	Cross sectional	3	Mean: 43.09 Range: 26–60	HTN; retinopathy; tinnitus; vestibular disorder; albuminuria	Unspecified	PTA; tympanometry; HFA	Collaborative healthcare efforts; preventative care
Pillay et al. [187]	Cross sectional	3	Unspecified	HTN; retinopathy; tinnitus; vestibular disorder; HIV; low-density lipoprotein; total cholesterol; low high-density lipoprotein cholesterol; obesity	Audiologist	PTA	Screening; hearing check-ups
Praneetha et al. [188]	Cross sectional	3	Unspecified	None provided	Unspecified	PTA; tympanometry; acoustic reflex testing	None
Rance et al. [189]	Cross sectional	3	Range: 35–61	Retinopathy; neuropathy	Unspecified	PTA; ABR; DPOAE	Screening
Rani & Kumar [69]	Cross sectional	3	Mean: 54.68 Range: 30–77	Tinnitus	Unspecified	PTA	Screening
Reddy & Sumatha [72]	Cross sectional	3	Mean: 56.4 Range: 30–70	HTN	Unspecified	PTA; otoscopy	Screening; hearing check-ups; early intervention; preventative care; early referral; collaborative healthcare efforts
H. Ren et al. [190]	Cross sectional	3	Mean: 50.68	HTN; retinopathy; neuropathy	Unspecified	PTA	None
J. Ren et al. [191]	Cross sectional	3	Mean: 40	Retinopathy; peripheral neuropathy; microalbuminuria; macroalbuminuria; hypercholesterolemia; hypertriglyceridemia; high low-density lipoprotein	Unspecified	PTA; ABR; TEOAE; DPOAE; EOAE	None
J. Ren et al. [192]	Cross sectional	3	Mean: 56.4	Retinopathy; nephropathy; peripheral neuropathy; atherosclerosis in carotid artery	Otolaryngologist	PTA; DPOAE	Preventative care
Roy et al. [193]	Prospective	3	Mean: 51.59 Range: 25–75	HTN; retinopathy; vestibular disorder; neuropathy; nephropathy	Unspecified	PTA; speech audiometry; tympanometry	None
Sakuta et al. [22]	Cross sectional	3	Mean: 52.9 Range: 51–59	HTN; obese; overweight; hypercholesterolemia; hypertriglyceridemia	Unspecified	PTA	None
Samelli et al. [194]	Cross sectional	3	Mean: 57.4 Range: 35–74	HTN; dyslipidemia	Unspecified	PTA; speech audiometry; tympanometry	None
Sanju et al. [195]	Cross sectional	2	Unspecified	None provided	Audiologist	PTA; ABR; speech audiometry; tympanometry; acoustic reflex testing	None
Sareen et al. [52]	Cross sectional	3	Mean: 52.3 Range: 32–74	None provided	Audiologist	PTA; ABR; DPOAE	Screening; hearing check-ups; risk reduction
Sasso et al. [196]	Cross sectional	4	Mean: 48.4 (study 1) 51.6 (study 2)	Retinopathy; peripheral neuropathy; nephropathy	Unspecified	PTA; ABR; tympanometry	None
Schade et al. [197]	Prospective	3	Mean: 55.4	HTN; hyperlipidemia; hyperglycemia	Audiologist	PTA	None
Sekaran et al. [198]	Observational	3	Range: 31–40	None provided	Unspecified	PTA; HFA; tuning fork	None
Seo et al. [199]	Retrospective	3	Mean: 60.5	HTN; tinnitus; vestibular disorder; cardiovascular disease	Unspecified	PTA	None
Shafiepour et al. [200]	Cross sectional	3	Unspecified	HTN	Unspecified	PTA; speech audiometry; otoscopy	None
Shah et al. [201]	Prospective	3	Unspecified	None provided	Unspecified	TEOAE; DPOAE; EOAE	Preventative care
Shaikh et al. [202]	Case–control	3	Range: 20–40	None provided	Unspecified	PTA	Screening
D.R. Sharma et al. [203]	Cross sectional	3	Range: 20–50	Retinopathy; vestibular disorder; neuropathy; nephropathy	Unspecified	PTA	None
R. Sharma et al. [204]	Cross sectional	3	Mean: 44.28 Range: 28–49	Neuropathy; chronic kidney disease; retinopathy	Unspecified	PTA; ABR; otoscopy; tuning fork	None
Y. Sharma et al. [205]	Cross sectional	3	Range: 40–50	None provided	Unspecified	PTA; ABR; tympanometry; tuning fork	None
Sheikhzadeh et al. [206]	Case–control	3	Unspecified	None provided	Unspecified	PTA; ABR; tympanometry; otoscopy	None
Shetty et al. [30]	Cross sectional	3	Mean: 47.87 Range: 40–55	None provided	Unspecified	PTA; DPOAE	Early referral; preventative care
Shin et al. [207]	Cross sectional	1	Unspecified	Chronic kidney disease; retinopathy or other vision disorders; stroke history; smoking; noise exposure; serum cholesterol; estimated glomerular filtration rate; HTN; smoking	Otolaryngologist	PTA	None
Shiralkar et al. [208]	Cross sectional	3	Range: 30–50	None provided	Unspecified	PTA; otoscopy	None
Sieger et al. [209]	Cross sectional	3	Range: 8–21 *	Retinopathy; hyperlipidemia; neuropathy	Unspecified	PTA; speech audiometry; tympanometry; acoustic reflex testing; CAEP	None
Snashall [210]	Cross sectional	3	Mean: 27 Range: 14–42 *	Ischemic heart disease; peripheral neuropathy; peripheral vascular disease; diabetic retinopathy (two); hypophysectomy for uncontrolled DM (one).	Unspecified	acoustic reflex testing	None
Solanki et al. [211]	Observational	3	Range: ≤60	None provided	Unspecified	PTA; otoscopy; tuning fork	Hearing check-ups
Sommer et al. [212]	Cross sectional	1	Unspecified	HTN; tinnitus; hyperacusis; chronic middle ear infection; ischaemic heart disease; cholesterol concentration; renal function	Research nurses	PTA; otoscopy; HFA	Follow-up hearing assessment; preventative care
Spankovich et al. [213]	Cross sectional	3	Mean: 22.75 Range: 18–28	None provided	Unspecified	PTA; ABR; tympanometry; acoustic reflex testing; TEOAE; DPOAE; EOAE; otoscopy	None
Spankovich et al. [62]	Cross sectional	3	Mean: 22.6 Range: 18–28	None provided	Unspecified	PTA; tympanometry; acoustic reflex testing; TEOAE; DPOAE; EOAE	None
Srinivas et al. [214]	Cross sectional	3	Range: 31–65	None provided	Unspecified	PTA; tuning fork	None
Sugimoto et al. [215]	Cross sectional	3	Unspecified	HTN; chronic kidney disease; retinopathy; hyperlipidemia, cerebrovascular accident, cardiovascular disease, diabetic neuropathy	Laboratory technicians	PTA; DPOAE; ASSR	None
Sushil et al. [216]	Cross sectional	3	Mean: 38.9 Range: 35–50	None provided	Unspecified	ABR	None
Swaminathan et al. [217]	Cross section swas nal	3	Range: 40–50	Hyperlipidaemia; high total cholesterol; high triglycerides; high LDL levels	Unspecified	PTA; tuning fork;	None
Takkar et al. [218]	Cross sectional	3	Unspecified	None provided	Unspecified	ABR	None
Tamil Selvan et al. [43]	Cross sectional	3	Unspecified	None provided	Unspecified	PTA	Screening
Tay et al. [219]	Cross sectional	3	Mean: 53.3 Range: 19–80	Retinopathy	Unspecified	PTA	None
Thimmasettaiah et al. [220]	Cross sectional	3	Unspecified	Retinopathy; neuropathy; nephropathy	Unspecified	PTA	None
Tiwari et al. [221]	Cross sectional	3	Unspecified	HTN	Unspecified	PTA; tuning fork	None
Uchida et al. [70]	Cross sectional	1	Range: 40–86	None provided	Unspecified	PTA; HFA	Screening
Vergou et al. [222]	Cross sectional	3	Mean: 41.8 (T1DM) 60.9 (T2DM)	HTN; cardiovascular disease	Unspecified	PTA; tympanometry; DPOAE; otoscopy	None
Vicente-Herrero et al. [223]	Cross sectional	3	Mean: 46	None provided	Unspecified	HFA	None
Vignesh et al. [44]	Cross sectional	3	Range: 20–40	None provided	Unspecified	PTA; HFA	Screening
Vinay Kiran et al. [224]	Cross sectional	3	Mean: 48.05 (DM good glycemic control) 49.3 (DM poor glycemic control)	None provided	Audiologist	PTA	None
Vinay Kiran & Ranganath [225]	Cross sectional	3	Mean: 50.1 Range: 40–60	None provided	Unspecified	ABR; tuning fork	Screening
Virtaniemi et al. [226]	Cross sectional	3	Mean: 25.5	None provided	Unspecified	PTA; ABR; speech audiometry; otoscopy; tuning fork	None
Virtaniemi et al. [227]	Cross sectional	3	Mean: 31.4 Range: 20–40	Retinopathy; nephropathy	Unspecified	PTA; ABR; speech audiometry	None
Weng et al. [228]	Retrospective	3	Mean: 60.1 Range: 35–84	HTN; hyperlipidemia	Unspecified	PTA	None
Wilson et al. [229]	Cross sectional	3	Mean: 49.7 (Male) 61.7 (Female)	Retinopathy	Unspecified	PTA; speech audiometry	None
Yadav & Yadav [230]	Cross sectional	3	Mean: 42.06 Range: 18–60	None provided	Unspecified	PTA	None
Yadav et al. [231]	Cross sectional	3	Mean: 50.83 Range: 35–65	Vestibular disorder	Unspecified	ABR; EOAE; tuning fork; ASSR	Screening; preventative care; collaborative healthcare efforts
Zivkovic-Marinkov et al. [232]	Prospective	3	Range: 40–60	None provided	Unspecified	PTA; ABR; TEOAE; EOAE	None

Note. * Indicates studies that included a pediatric population. ^1^ CEBM: Centre for Evidence-Based Medicine Levels. ^2^ Acronyms defined: ABR: auditory brainstem response; CAEP: cortical auditory evoked potentials; DPOAE: distortion product otoacoustic emissions; EOAE: evoked otoacoustic emissions; HFA: high-frequency audiometry; PTA: pure-tone audiometry; SFOAE: stimulus frequency otoacoustic emissions; SIN: speech in noise; TEOAE: transient evoked otoacoustic emissions. ^3^ Recommendations defined: Audiological evaluation: assessment of the auditory system using objective measures; Follow-up: ongoing monitoring or reassessment of a patient’s HL over time; Hearing check-ups: routine or regular comprehensive monitoring practices, regardless of whether HL has been identified; Hearing screening: test to identify individuals who may have HL; Metabolic assessment: evaluation of metabolic health (e.g., assessing blood glucose levels, HbA1c, lipids); Preventative care: strategies and interventions such as protocol modifications, routine screening questions, integration of hearing assessments into standard care, and education and awareness efforts aimed at reducing the risk or impact of disease and its complications; Risk reduction: management strategies (e.g., blood glucose control, addressing early physiological changes) to lower the likelihood, progression, or severity of HL.
