# Supplementary material for: Hearing Health Monitoring in Adults Living with Diabetes: A Scoping Review to Inform Integrated Care Pathways
Source: Audiol Res. 2026 Sep 10;16(5):134. doi: 10.3390/audiolres16050134 (PMC13600158; doi:10.3390/audiolres16050134)
Supplement: Supplementary file 1 [file audiolres-16-00134-s001.zip › Supplement File S1_Data Charting Tool.pdf]

**Supplement A.** Data charting tool.

| Factor | Extraction details |
|---|---|
| Research topic? | Description, including study design. |
| Participant group? | Number of total participants, group specific sample size, age range, mean age of all groups within study, average age of diagnosis for diabetes. |
| Other conditions of interest purposefully studied within this study? | Healthy controls, controlled diabetes, uncontrolled diabetes, hypertension, chronic kidney disease, retinopathy or other vision disorders, other. |
| Which type of diabetes was examined? | Type 1 diabetes, type 2 diabetes, gestational diabetes, maturity-onset diabetes of the young, neonatal diabetes, other. |
| What type of treatment did the participants receive for their diabetes as part of their personal care plan? Did they receive treatment for diabetes as part of the research study, if so elaborate what that was. Specify if participant groups received different treatments. | Description (insulin, medication, etc.) |
| What professional identified the hearing loss and at what point was it identified (before or during study)? | Description (audiologist, otolaryngologist, etc.) |
| What professionals were involved in the care of the patient and at what point in their care journey(before, after, during the study etc.)? | Description (audiologist, otolaryngologist, etc.). |
| Indicate the professionals on the research team as well as their academic/departmental affiliations | Description. |
| Please indicate the role of each professional on the team. | Description (auditory examination, etc.). |
| Please indicate the role of each professional in the identification and/or management of hearing loss within the study. | Description. |
| Please indicate the management strategies for diabetes and the associated professional if applicable | Description. |
| In which settings is hearing loss being identified among individuals with diabetes? | Hospital, outpatient rehabilitation center, inpatient rehabilitation center, school, community setting, community clinic, within the study, not specified, other. |
| What clinical tools are used in the identification of hearing loss in individuals with diabetes? | Pure-tone audiometry, speech audiometry, tympanometry, acoustic reflex testing, TEOAE, DPOAE, SPOAE, ABR, ECochG, high-frequency audiometry, cortical auditory evoked potentials, ASSR, Stenger test, other, |
| Describe the test battery (include citation if available) used in the identification of hearing loss. | Description (equipment, frequency ranges, etc.). |

| | |
|---|---|
| Indicate how the results of the study informed the next steps in the care of the patient, if applicable. | Description (e.g., moderate hearing loss in high frequencies indicating the need for yearly monitoring) |
| What questionnaires or self-assessment tool was used to identify hearing loss (if applicable)? | Description (HHIE, Yes/No question, No questionnaire was provided, other). |
| Did the patient receive care from an audiologist outside of the research study itself? | Yes/No. If yes, indicate how the patient came to receive audiology care (outside of just the research study). (Referral, frontline access, other) |
| If you selected referral, indicate what triggered referral to the audiologist? | Description. |
| Who completed the referral to audiologist? | Description. |
| What was the role of the audiologist in the care of the patient? | Description. |
| In addition to an audiologist, what other healthcare professionals was this patient referred to and who completed the referral (if indicated)? | Description. |
| Indicate what triggered referral to other health professionals. | Description. |
| What were the risk factors associated with hearing loss in diabetics and provide any explanations for how selected risk factors impacted hearing loss? | Description (duration of diabetes, age, sex, etc.). |
| Was a recommendation made regarding audiology care, follow-up hearing assessment, treatment options, hearing assistive technologies, and/or hearing check-ups (e.g., to support early detection and intervention or to monitor for progression)? | Yes/No. If yes, please select the recommendations (follow-up hearing assessment, screening, hearing assistive technology, hearing check ups, counselling, other.) |
| If you selected hearing assistive technology please describe. | Description. |
| Were management strategies suggested around caregiver education and/or family counselling (or other)? | Description. If yes, indicate what the management strategies were. |
| Describe any reported impacts of hearing loss for the study participants. | Description. |
| Was follow up testing completed (related to hearing loss)? | Yes/No/Not sure. If yes, describe the follow-up care routine that was recommended (e.g. how often?) |
| What were the key findings related to hearing functioning among diabetic patients? | Description. |
| Indicate the key findings of the study. | Description. |
